# Bromide of Potassium in after Pains

**Published:** 1871-03

**Authors:** R. B. Anderson

**Affiliations:** Roswell, Ga.


### BROMIDE OF POTASSIUM IN AFTER-PAINS.

BY R. B. ANDERSON, M. D., OF ROSWELL, GA.

I was called on the 20th of this inst. (January,) at 6 o'clock, A. M, to see Mrs. ——, of this place, in her 6th labor. One hour and a half after my arrival, she was safely delivered of her sixth son. The Placenta was thrown off in ten minutes after the delivery of the child, and as usual with her, she began having the severest after-pains I ever witnessed, except in her two former labors—one in December, 1868, and the other in December 1869. Having then tried several remedies, none of which gave relief, I determined to give her a different treatment on this occasion.

Twenty minutes after the removal of the placenta, I gave her opii gr. 1, bromide of potassium gr. xx. In one hour the brom. potassium was repeated with one grain of gum camphor, and ordered to be given every hour through the day. I called to see her at 8 o'clock, P. M., found her doing well—had passed the day with much less pain than usual, with her. The dose was then reduced to half the quantity. (brom. potass. x grains, camphor ½ gr.) to be given every hour; 10 o'clock A. M., the 21st, I saw her again—pains rather harder than they had been through the night—continued the medicine. At 5 P. M., saw her again—she had rested better through the day—continued the medicine. 10 o'clock, A. M., the 22d, quite comfortable—rested well through the night—took no medicine after 10 o'clock, at night, until 7 o'clock, A. M. The 23d I saw her at 8 o'clock, A. M., ordered the medicine every three hours. 2 o'clock, P. M. doing well—ordered the medicine given at intervals of four hours—saw her on the 24th, feeling well. Treatment discontinued—had no further trouble.

---

Colic.—From ten to twenty minims of chloroform, combined with one or two drachms of comp. tr. of camphor—or acetate morphine, will be found an excellent combination in relieving the pain of colic.

Choral in Hernia.—Dr. B. W. Richardson thinks choral will prove valuable in strangulated hernia, in consequence of its producing complete muscular relaxation.

